# Exploring Comorbid Addictive Symptoms in Gambling Disorder: The Role of Emotion Regulation, Alexithymia, and Social Support

**DOI:** 10.1007/s10899-026-10481-8

**Published:** 2026-03-07

**Authors:** Ismael Muela, Gema Aonso-Diego, Ana Estévez

**Affiliations:** Department of Psychology, Faculty of Health Sciences, University of Deusto, Bilbao, 48007 Spain

**Keywords:** Gambling, Behavioral addictions, Comorbidity, Emotion regulation, Alexithymia, Social support

## Abstract

Gambling disorder (GD) often co-occurs with other addictive behaviors, but the psychological mechanisms underlying this comorbidity remain poorly understood, particularly in clinical samples. This cross-sectional study examined whether alexithymia, dispositional emotion regulation strategies, and perceived social support help explain or moderate the association between problem gambling severity and additional addictive behaviors in 90 individuals diagnosed with GD and currently receiving treatment. Participants completed validated measures of addictive behaviors, emotion regulation (adaptive and maladaptive strategies), alexithymia, and perceived social support. A theory-informed, stepwise analytical approach was followed, including multiple linear regression, moderation, and mediation analyses. Problem gambling severity significantly predicted the presence of additional addictive behaviors. Maladaptive emotion regulation strategies (MERS), difficulty identifying feelings (DIF), and low social support were independently associated with comorbid addictive symptoms. Social support moderated the association between problem gambling severity and comorbidity, such that the link was weaker at higher support levels. The mediation model involving MERS showed a potential indirect effect, but this effect did not reach conventional statistical significance. No significant mediation was found for DIF. These findings highlight the relevance of emotional and interpersonal functioning in the broader addictive profile of individuals with GD. Clinically, they suggest that routine assessment of emotion regulation and perceived social support in GD services may help identify patients at greater risk for broader addictive comorbidity and inform more integrated interventions. Future studies should explore moderated mediation models to clarify these mechanisms and inform more targeted interventions.

## Introduction

Gambling disorder, recognized as a behavioral addiction in the *Diagnostic and Statistical Manual of Mental Disorders (*Fifth Edition, Text Revision*;* DSM-5-TR; American Psychiatric Association, 2022), is characterized by a persistent and recurrent pattern of gambling that leads to significant distress or impairment. Beyond its toll on the individual, gambling disorder often has devastating long-term consequences at the financial, emotional, and social levels, affecting not only the person who gambles but also their family members, friends, work colleagues, and broader social environment (Azemi et al., 2023; Dowling et al., 2016; Hautamäki et al., 2025; Hing et al., 2022; Lind et al., 2022). In addition to its wide-reaching impact, this disorder frequently co-occurs with other addictive behaviors, including both substance-related and other behavioral addictions (Abdollahnejad et al., 2014; Bischof et al., 2013; Cowlishaw et al., 2014; Dowling et al., 2015; Elman et al., 2016; Grant & Kim, 2003; Håkansson et al., 2018; Leino et al., 2023; Petry et al., 2005). While this comorbidity has been widely documented, recent longitudinal evidence suggests that gambling problems may not only coexist with but also precede and predict the onset of other addictions, particularly among young or clinically vulnerable populations (Afifi et al., 2016; García‑Maza et al., 2025; Leino et al., 2023). Still, findings are not entirely consistent, as some studies indicate that substance use problems may precede gambling disorder (Dowling et al., 2019) and concurrent decreases in gambling problems and other addictive behaviors have also been observed (e.g., Kim et al., 2024).

The link between problem gambling severity and the emergence of additional addictive behaviors may be shaped by both biological mechanisms and transdiagnostic psychological vulnerabilities. Among the biological processes, dopaminergic sensitization and cross-sensitization have also been proposed to account for the overlap between gambling disorder and other addictive behaviors (Anselme & Robinson, 2019). According to the incentive sensitization theory, repeated exposure to addictive stimuli enhances dopaminergic reactivity to reward-related cues, thereby increasing incentive salience and the motivational drive to seek such rewards (Robinson & Berridge, 2008). Cross-sensitization phenomena have been observed between drugs and gambling (Boileau et al., 2013; Zack et al., 2014), suggesting that neural sensitization to one type of rewarding experience may facilitate the pursuit of others. Similarly, stress-related activation of dopaminergic circuits has been proposed to potentiate gambling behavior through shared sensitization processes (Biback & Zack, 2015; Zack et al., 2020). In this view, biological sensitization processes may potentiate the psychological vulnerabilities that sustain the co-occurrence of gambling and other addictive behaviors.

Beyond simple co-occurrence, research suggests that comorbidity between gambling disorder and other addictive and psychiatric conditions is both common and clinically meaningful. Meta-analytic and narrative reviews consistently report high rates of co-occurring substance use disorders (Armoon et al., 2025; Crockford & el-Guebaly, 1998; Dowling et al., 2015; Lorains et al., 2011; Rash et al., 2016; Sharma & Weinstein, 2025), mood and anxiety disorders (Armoon et al., 2025; Crockford & el-Guebaly, 1998; Dowling et al., 2015; Lorains et al., 2011; Rash et al., 2016; Sharma & Weinstein, 2025), and other behavioral addictions (Lorains et al., 2011; Tang et al., 2020) among individuals with GD, particularly in treatment-seeking samples, and indicate that comorbid profiles tend to be associated with greater gambling severity and poorer treatment outcomes (Lorains et al., 2011; Rash et al., 2016; Sharma & Weinstein, 2025; Tang et al., 2020; Yau & Potenza, 2015). Conceptually, these findings are consistent with the view that GD and other addictive disorders may be understood as partially overlapping manifestations of broader addictive liabilities, rather than entirely independent conditions (Rash et al., 2016). In this framework, treating other substance-related and behavioral addictions as comorbidities of GD is both clinically relevant and theoretically consistent with dimensional and transdiagnostic models of addiction (Rash et al., 2016; Sharma & Weinstein, 2025; Szerman et al., 2020).

At the psychological level, difficulties in emotion regulation (Buen & Flack, 2022; Dingle et al., 2018; Estévez et al., 2022, 2023; Garke et al., 2021; González-Roz et al., 2024; Jauregui et al., 2023; Navas et al., 2016; Ruiz de Lara et al., 2019; Stellern et al., 2023; Theodorou et al., 2025), elevated levels of alexithymia (Bonnaire et al., 2017; Honkalampi et al., 2022; Jauregui et al., 2023; Kun et al., 2023; Marchetti et al., 2019; Noël et al., 2018; Topino et al., 2024), and low perceived social support (Afifi et al., 2016; Bickl et al., 2024; Mancini, 2025; Oei & Gordon, 2008; Petry & Weiss, 2009; Uçur & Dönmez, 2021) have each been independently associated with gambling disorder as well as with a range of substance-related and other behavioral addictions. However, their combined contribution and their potential roles as mediating or moderating mechanisms in this relationship remain understudied, particularly in clinical settings.

Emotion dysregulation has been widely identified as a transdiagnostic factor involved in the development and maintenance of various psychological disorders, including addictive behaviors (Buen & Flack, 2022; Cludius et al., 2020; Jara-Rizzo et al., 2019; Navas et al., 2017; Rogier & Velotti, 2018). In the context of gambling, difficulties in managing intense affective states often manifest as a reliance on maladaptive strategies such as rumination, catastrophizing, or self-blame (strategies that not only exacerbate emotional distress but are also consistently associated with greater problem gambling severity; Estévez et al., 2023; González-Roz et al., 2024; Navas et al., 2016; Ruiz de Lara et al., 2019). Moreover, recent findings in clinical populations suggest that even strategies typically considered adaptive (such as acceptance or positive reappraisal) can be linked to increased severity when applied ineffectively or in inappropriate contexts (Estévez et al., 2024). Thus, emotion dysregulation may not only play a role in the escalation of gambling behavior but could also contribute to the emergence of comorbid addictive patterns. Beyond gambling, emotion dysregulation has also been consistently linked to other substance use disorders, as well as to putative or established behavioral addictions, and has been described as a core transdiagnostic risk factor underlying the development of substance use, addiction, and comorbid psychopathology (Garke et al., 2021; Hand et al., 2024; Shadur & Lejuez, 2015; Spence & Courbasson, 2012; Stellern et al., 2023).

Closely linked to emotion regulation difficulties and addictive behavior (including gambling disorder) is the construct of alexithymia, defined as the difficulty in identifying and describing one’s emotional states (Marchetti et al., 2019; Morie et al., 2016; Orsolini, 2020). A growing body of evidence indicates that individuals with gambling disorder score significantly higher in alexithymia than non-gamblers, particularly on the subdimensions "Difficulties in Identifying Feelings" (DIF) and "Difficulties in Describing Feelings" (DDF; Bonnaire et al., 2017; Jauregui et al., 2023; Noël et al., 2018). These deficits appear to be especially pronounced among strategic gamblers, for whom alexithymia has been associated with higher clinical severity (Bonnaire et al., 2017). Meta-analyses and systematic reviews have further highlighted that alexithymic traits are more prevalent in both community and clinical problem gamblers, correlate with problem gambling severity, and may interact with other vulnerability factors such as dysfunctional beliefs or certain personality traits (Marchetti et al., 2019). Additionally, alexithymia has been proposed as a psychological mechanism through which some individuals may use gambling as a strategy to enhance emotional arousal or to suppress intense negative states (Noël et al., 2018; Topino et al., 2024). While some studies in adolescents or low-risk gamblers have failed to find consistent differences (Estévez et al., 2022), findings in clinical samples tend to be robust and convergent. In addition, alexithymia has been associated with a range of substance-related and behavioral addictions beyond gambling (Marchetti et al., 2019; Morie et al., 2016; Orsolini, 2020; Palma-Álvarez et al., 2021). Studies in individuals with substance use disorders indicate that higher alexithymia scores are linked to greater psychiatric comorbidity and more severe psychological symptoms (e.g., depression and anxiety; Palma-Álvarez et al., 2021), which is consistent with the view that difficulties in identifying and describing emotions may play a transdiagnostic role and could help account, at least in part, for the overlap observed between gambling disorder and other addictive conditions.

Beyond emotional vulnerabilities, perceived social support has been consistently identified as a potential protective factor against the development and maintenance of addictive behaviors (Akbari et al., 2023; Atadokht et al., 2015; Jodis et al., 2023; McCollum et al., 2024; Mo et al., 2018). In the case of gambling disorder specifically, low levels of support are associated not only with a higher risk of gambling initiation and participation (Bickl et al., 2024; Kim et al., 2024), but also with a greater clinical severity (Marinaci et al., 2021; Savolainen et al., 2019), more gambling-related problems (Bickl et al., 2024; Koompah et al., 2024), poorer treatment outcomes (Petry & Weiss, 2009), worse prognosis, and shorter periods of abstinence (Oei & Gordon, 2008). Longitudinal studies reinforce this pattern, indicating that social support predicts lower problem gambling severity over time (Mancini, 2025) and may serve as a key protective factor, particularly among young men (Bickl et al., 2024). Moreover, recent findings suggest that social support may also mitigate the emotional and psychosocial harm experienced both by individuals who gamble and their significant others (Tulloch et al., 2025), and that a distinct sense of belonging, such as that fostered in mutual-help groups, may reduce gambling urges (Penfold & Ogden, 2024). Low perceived social support has likewise been implicated in alcohol and substance use problems and in other addictive behaviors (Akbari et al., 2023; Atadokht et al., 2015; Beitchman et al., 2005; Mo et al., 2018; Warren et al., 2007). Studies in individuals with substance use and co-occurring psychiatric disorders indicate that higher levels of social support are associated with better mental health and reduced substance use over the course of treatment (Jarnecke et al., 2022; Warren et al., 2007), and adolescent data suggest that low family support is particularly characteristic of youth with substance abuse and broader adjustment problems (Beitchman et al., 2005). In addition, recent findings from young adult samples show that perceived social support tends to be lower among individuals who report multiple co-occurring addictions compared with those with a single or no addiction (Tomei et al., 2025), suggesting that social support is closely intertwined with the presence of addictive comorbidity, even though its specific role in shaping comorbid profiles remains to be clarified.

### The Present Study

Despite the extensive literature linking alexithymia, emotion dysregulation, and low social support to addictive behaviors, their combined role in the relationship between problem gambling severity and additional addictions in individuals with gambling disorder remains largely unexplored, particularly in clinical contexts. This gap is especially relevant given the high rates of comorbidity between gambling disorder and other addictive conditions (e.g., Dowling et al., 2015) and the need to identify the psychological mechanisms underlying this overlap. Moreover, much of the existing research relies on general or subclinical populations (e.g., Abdollahnejad et al., 2014; Bischof et al., 2013; Håkansson et al., 2018), limiting the applicability of findings to individuals already experiencing functional impairment. To our knowledge, no previous studies have systematically examined whether difficulties in emotion regulation, alexithymia, and perceived social support mediate or moderate the association between problem gambling severity and broader addictive symptomatology in treatment-seeking individuals with gambling disorder.

The present study was conducted in a sample composed exclusively of individuals diagnosed with gambling disorder who were currently receiving treatment. Focusing on a treatment-seeking clinical group allowed us to examine these mechanisms in a context of established impairment and service use.

The study had three main aims. First, we examined whether problem gambling severity was associated with the presence of additional addictive symptoms. In line with our operationalization, this outcome was defined as a composite index of comorbid addictive symptoms derived from a screening measure, reflecting risk indicators of additional addictive behaviors rather than formal comorbid diagnoses. Second, we tested whether alexithymia, cognitive emotion regulation strategies (both adaptive and maladaptive), and perceived social support explained additional variance in comorbid addictive symptoms beyond that accounted for by problem gambling severity. We expected that individuals with higher alexithymia scores, greater use of maladaptive strategies, and lower levels of perceived support would report a greater number of additional addictive symptoms. Third, we explored whether these psychological factors moderated or mediated the association between problem gambling severity and broader addictive symptomatology. Given the limited prior work testing these specific mechanisms in clinical samples, all analyses were considered exploratory.

## Method

### Participants and Procedure

The sample consisted of 90 individuals undergoing treatment for gambling-related problems in various associations affiliated with the Spanish Federation of Rehabilitated Gambling Addicts (Federación Española de Jugadores de Azar Rehabilitados; FEJAR). Data collection took place between November 2023 and July 2024. The mean age of participants was 47.16 years (SD = 14.89), with ages ranging from 21 to 76 years. Regarding gender, 28.1% were women and 71.9% were men. The number of valid responses varied slightly depending on the scale considered, ranging from 73 to 89 cases, due to some missing data. In this sense, for inferential analyses, regression and moderation models were estimated using casewise (listwise) deletion as implemented in JASP, whereas correlation analyses were based on pairwise deletion. In addition, the key regression, moderation, and mediation models were re-estimated using full information maximum likelihood (FIML) in R (lavaan) to examine the robustness of the findings under a likelihood-based approach to missing data (see Supplementary Appendix S3). Descriptive statistics for the main study variables are presented in Table 1, and a detailed description of the sociodemographic characteristics of the sample is provided in Supplementary Table S1.

**Table 1:** Descriptive statistics for main study variables

Variable	*n*	*M*	*SD*	Range
Problem gambling severity	73	2.82	1.23	0–4
CAS	77	0.77	0.63	0–3.43
AERS	78	15.91	2	11.4–20
MERS	79	12.81	2.66	5.5–20
DIF	82	22.1	8.8	7–42
Social Support	76	78.15	18.06	19–95
Age	89	47.16	14.89	21—76

*CAS*, Comorbid addictive symptoms; *AERS*, Adaptive emotion regulation strategies; *MERS*, Maladaptive emotion regulation strategies; *DIF*, Difficulties in Identifying Feelings

The questionnaires were administered in paper format and in person, primarily at the participating associations. In most cases, psychologists specialized in behavioral addictions visited the centers to conduct the assessment; in other cases, the instruments were administered by the professionals responsible for treatment at each association. Participation was voluntary, anonymity was guaranteed, and all participants signed an informed consent form prior to the study, after receiving a detailed explanation of the procedure and having the opportunity to ask questions. The assessment was completed in a single session.

### Measurements

*MultiCAGE CAD-4 (*Pérez et al., 2007*).* The MultiCAGE CAD-4 is a brief screening tool designed to detect the risk of addictive behaviors across eight domains: problematic alcohol use, illegal drug use, gambling, excessive Internet use, excessive video gaming, compulsive sexual behavior, compulsive buying, and eating disorders. Each subscale consists of four dichotomous (yes/no) items that assess (1) subjectively experienced craving, (2) complaints from relatives or acquaintances, (3) guilt, shame, or denial regarding the behavior, and (4) self-reported compensatory or control efforts.

In the present study, the gambling subscale was used as a measure of problem gambling severity. Additionally, a composite score was calculated to represent comorbidity with other addictive behaviors by averaging the scores of the remaining seven subscales. Because the MultiCAGE CAD-4 is a brief screening tool, this composite index reflects the presence of risk indicators or symptoms of multiple substance-related and behavioral addictions rather than formal comorbid diagnoses. Although these subscales tap into conceptually distinct behaviors (e.g., alcohol and drug use, compulsive buying, compulsive sexual behavior, eating-related problems), they showed high internal consistency as a composite in this sample (see next paragraph), suggesting a shared dimension of addictive symptomatology. At the same time, this approach does not capture potential behavior-specific differences and should therefore be interpreted as an index of overall addictive symptom load rather than of any particular comorbid condition. The use of the mean, rather than the sum, allowed us to maintain the same metric (0–4) as the gambling subscale, facilitating direct comparison and minimizing the influence of the number of subscales on score interpretation.

In this sample, internal consistency was adequate for the full scale (Cronbach’s α =.825). The gambling subscale showed acceptable reliability (α =.669), while the composite score of the remaining subscales demonstrated high internal consistency (α =.822).

*Cognitive Emotion Regulation Questionnaire (CERQ; *Garnefski et al., 2001)*. Spanish adaptation by *Domínguez-Sánchez et al., 2013*).* The CERQ is a 36-item self-report instrument that assesses the use of specific cognitive strategies in response to negative or stressful life events. Items are rated on a 5-point Likert scale ranging from 1 (almost never) to 5 (almost always). The questionnaire includes nine subscales, each composed of four items. Five of these subscales reflect theoretically adaptive strategies: Acceptance, Refocus on Planning, Positive Refocusing, Positive Reappraisal, and Putting into Perspective. The remaining four reflect theoretically maladaptive strategies: Self-blame, Other-blame, Rumination, and Catastrophizing.

In line with previous research (e.g., Ackermans et al., 2024; Estévez et al., 2022; Navas-Casado et al., 2023; Shahrajabian et al., 2024; Vanderhasselt et al., 2014; Zhang et al., 2020), two composite scores were computed: an adaptive cognitive emotion regulation score (AERS), calculated as the mean of the five adaptive subscales; and a maladaptive cognitive emotion regulation score (MERS), calculated as the mean of the four maladaptive subscales. These scores capture individual differences in regulatory tendencies that have been associated with emotional well-being and psychological vulnerability, respectively.

In the present sample, internal consistency was acceptable to good for both composites: α =.747 for AERS and α =.807 for MERS. The overall reliability of the full CERQ scale was high (Cronbach’s α =.830).

*Toronto Alexithymia Scale – 20 items (TAS-20; *Bagby et al., 1994*); Spanish adaptation by *Martínez Sánchez, 1996*)*. The TAS-20 is a 20-item self-report questionnaire developed to assess alexithymia across three dimensions: (1) DIF, referring to problems in recognizing and distinguishing emotional states from bodily sensations; (2) DDF, which reflects challenges in verbalizing emotions and using emotional language; and (3) Externally-Oriented Thinking (EOT), a cognitive style marked by a focus on concrete external events over internal emotional experiences. All items are rated on a 6-point Likert scale ranging from 0 (strongly disagree) to 5 (strongly agree).

In the present study, only the DIF and DDF subscales were used, as these two dimensions are most directly related to emotional awareness and expressive functioning, and have been more consistently linked to addictive and dysregulated behaviors in the literature. Meta-analytic and systematic evidence indicates that DIF and DDF show stronger and more reliable associations with both substance use and behavioral addictions compared to the EOT dimension (Honkalampi et al., 2022; Kun et al., 2023; Marchetti et al., 2019). The DIF subscale consists of 7 items, and the DDF subscale includes 5 items.

Internal consistency in the current sample was excellent for DIF (Cronbach’s α =.892) and poor for DDF (α =.497).

Medical Outcomes Study Social Support Survey (MoS-SSS; Sherbourne & Stewart, 1991*; Spanish adaptation by *Revilla et al., 2005). The MOS-SSS is a 19-item self-report questionnaire designed to assess perceived availability of social support across four functional domains: emotional/informational support, tangible support, affectionate support, and positive social interaction. Respondents rate how often different kinds of support are available to them (e.g., “Someone to give you good advice about a crisis,” “Someone who hugs you”) using a 5-point Likert scale ranging from 1 (*none of the time*) to 5 (*all of the time*). Scores are typically averaged, with higher scores indicating greater levels of perceived support.

Following the original authors’ recommendations and prior empirical evidence (Sherbourne & Stewart, 1991), we used the overall composite score as a global indicator of perceived social support. This approach has been widely adopted in psychological and health research as a valid unidimensional measure.

In the present sample, internal consistency for the total scale was excellent (Cronbach’s α =.965).

### Ethics

All participants gave informed consent in writing before taking part in the study. Participation was entirely voluntary, and individuals were guaranteed both the anonymity and confidentiality of their responses. No form of compensation was offered for participation. Ethical approval was obtained from the ethics committee of the corresponding institution [(name and reference anonymized for review)], and the study was conducted following the principles of the Declaration of Helsinki.

### Statistical Analysis

Following a data-driven yet theory-informed approach, the analytic strategy proceeded sequentially: each step was informed by the results of the previous one and grounded in relevant theoretical frameworks. This approach allowed us to identify meaningful relationships in the data while avoiding arbitrary model testing and helped to reduce the risk of Type I error associated with multiple unplanned comparisons. Following a data-driven yet theory-informed approach, the analytic strategy was designed to proceed sequentially: each step was informed by the results of the previous one and grounded in relevant theoretical frameworks. This approach enabled us to identify meaningful relationships in the data without engaging in arbitrary model testing, thereby reducing the risk of Type I error associated with multiple unplanned comparisons. By combining empirical sensitivity with conceptual consistency, we aimed to maintain methodological transparency and analytical coherence throughout the study.

All analyses were conducted using JASP software (version 0.19.3; JASP Team, 2025). Prior to conducting inferential analyses, we examined the internal consistency of all measures (as previously reported in the Methods section). As noted, the DDF subscale of the TAS-20 showed poor reliability (Cronbach’s α =.497). Given this limitation, DDF was excluded from further analyses to ensure the robustness of the statistical models. Only the DIF subscale (α =.892) was retained as a measure of alexithymia.

Missing data were handled in two stages. First, at the scoring stage, composite and subscale scores were computed only when sufficient item-level information was available; when the items required for a given subscale or composite were incomplete, that specific score was set to missing, but other available scales for the same participant were retained. Second, at the modelling stage, missing data were handled by the statistical software (JASP), which applies casewise (listwise) deletion within each regression and moderation model, and pairwise deletion in the computation of correlation matrices.

Also, before proceeding with the main analyses, we verified that the key assumptions of multiple linear regression were reasonably met. Independence of residuals was confirmed with the Durbin-Watson test, and no evidence of multicollinearity was found (all VIFs < 2). Visual inspection of residual-versus-fitted plots indicated approximate linearity and homoscedasticity. Histograms and Q-Q plots of residuals showed no substantial departures from normality.

A stepwise analytical approach was adopted, grounded in prior theory and sequentially informed by the results of previous analyses. Having confirmed the reliability of the measures and the adequacy of the model assumptions, we then conducted a (hierarchical) multiple linear regression analysis to examine the psychological correlates of comorbid addictive symptoms. Predictors were entered in two blocks based on theoretical considerations. In the first step, a model including only problem gambling severity, age, and gender was tested. In the second step, all psychological variables of interest (i.e. AERS, MERS, DIF [alexithymia subscale], and perceived social support) were added to the model to assess their contribution to explaining additional variance in the outcome. This procedure allowed us to evaluate whether the psychological constructs were associated with the presence of other addictive behaviors beyond the effect of problem gambling severity.

Next, moderation analyses were conducted to assess whether any of the psychological variables significantly associated with the outcome moderate the relationship between problem gambling severity and comorbid addictive symptoms. These analyses were only conducted when the potential moderators had previously shown a significant association with the outcome variable (which was the case for all three constructs). For significant interaction terms, simple slopes analyses were conducted at low (− 1 SD), mean, and high (+ 1 SD) levels of the moderator to clarify the form of the interaction, and the interaction was graphically represented using predicted values of the outcome variable.

Finally, exploratory mediation models were estimated using the maximum likelihood method to assess whether the relationship between problem gambling severity and comorbid addictive symptoms was mediated by selected psychological variables. These models were only tested when the variables had shown a significant association with the outcome and did not exhibit significant interaction effects in the moderation analyses, which was the case for DIF (alexithymia) and MERS.

In this way, the progression from multiple regression to moderation and, where applicable, to mediation models was not arbitrary, but conditional on both the theoretical rationale outlined in the Introduction and the empirical pattern observed at each step. For all analyses, statistical significance was set at *p* <.05. Effect sizes and 95% confidence intervals were reported where appropriate. Additionally, to support future research and replication efforts, bivariate correlations among all study variables are reported in Supplementary Materials (Table S2).

Finally, given the relatively modest sample size, we also conducted a sensitivity power analysis using G*Power version 3.1.9.7 (Faul et al., 2009) to determine the minimum detectable effect sizes for the main regression and moderation models. For the hierarchical regression model including seven predictors and the smallest complete-case sample size (*N* = 73), the analysis was performed with α =.05 and power (1 – β) =.80. A similar sensitivity analysis was conducted for the moderation models including the interaction term (four predictors; *N* = 66).

Lastly, to address concerns about the handling of missing data, we conducted a series of additional full information maximum likelihood (FIML) re-analyses in R (version 2026.01.0); lavaan package). These robustness checks, which re-estimate the main regression, moderation, and mediation models under a likelihood-based missing-data approach, are described in Supplementary Appendix S3.

### Transparency and Openness

Materials and analysis code for this study are available by emailing the corresponding author.

## Results

### Regression Analyses

Results of the hierarchical multiple linear regression model examining whether problem gambling severity and psychological variables (i.e., MERS, AERS, DIF, and perceived social support) were associated with comorbid addictive symptoms, while controlling for age and gender, are summarized in Table 2.

**Table 2:** Linear regression model predicting comorbid addictive behaviors from gambling severity (controlled for age and gender)

Outcome	Predictor		*B*	*SE*	*β*	*t*	*p*	95% CI
Comorbid addictive symptoms	(Intercept)		0.450	0.324		1.388	.170	[–0.197, 1.097]
	Problem gambling severity		0.132	0.063	0.248	2.088	.041*	[0.006, 0.259]
	Age		−0.002	0.006	−0.048	−0.389	.698	[–0.014, 0.009]
	Gender		0.122	0.186		0.656	.514	[–0.014, 0.009]
	*R*^*2*^ = 0.074, *F* (3, 70) = 1.1772, *p* =.161							
Comorbid addictive symptoms	(Intercept)	0.135		0.779		0.174	.863	[–1.425, 1.696]
	Problem gambling Severity	0.144		0.064	0.258	2.242	.015*	[0.015, 0.273]
	MERS	0.067		0.033	0.272	2.021	.048*	[6.558 × 10^–4^, 0.133]
	AERS	0.003		0.037	0.010	0.086	.932	[−0.072, 0.078]
	Social support	−0.009		0.004	−0.246	−2.072	.043*	[−0.018, −3.157 × 10^–4^]
	DIF	0.019		0.009	0.256	2.075	.042*	[6.770 × 10^–4^, 0.038]
	Age	−0.008		0.006	−0.166	−1.422	.160	[−0.020, 0.003]
	Gender	−0.064		0.177		−0.359	.721	[−0.419, 0.292]
	*R*^*2*^ = 0.330, *F* (3, 65) = 4.076, *p* =.001							

Gender was coded as 0 = male, 1 = female. *B*, Unstandardized regression coefficient; *β*, Standardized regression coefficient; *SE*, Standard error; *CI*, Confidence interval. *p < 0.05. *AERS*, Adaptive emotion regulation strategies; *MERS*, Maladaptive emotion regulation strategies; *DIF*, Difficulties in Identifying Feelings

In the first step, a model including only problem gambling severity, age, and gender was tested. As shown in the upper section of Table 2, this model was not statistically significant*, F*(3, 67) = 1.77, *p* =.161, accounting for 7.4% of the variance (*R*^*2*^ =.074). However, problem gambling severity emerged as a significant predictor (β = 0.248, *t* = 2.088, *p* =.041), while age and gender did not contribute significantly to the model (*p-values* ≥.514).

In the second step, the full model, including all psychological variables, was tested. This model was statistically significant, *F*(7, 58) = 4.08, *p* =.001, explaining approximately 33% of the variance in comorbid addictive symptoms (*R*^*2*^ =.330, adjusted *R*^*2*^ =.249).

As shown in the lower section of Table 2, greater problem gambling severity (*β* = 0.258, *t* = 2.242, *p* =.029), higher use of MERS (β = 0.272, *t* = 2.021, *p* =.048), and DIF (β = 0.256, *t* = 2.075, *p* =.042) were all positively associated with the presence of additional addictive behaviors. In contrast, perceived social support was negatively associated with the outcome (β = −0.246, *t* = −2.072, *p* =.043). AERS, age, and gender were not significant predictors in the final model (*p-*values >.160).

### Moderation Analyses

To examine whether the relationship between problem gambling severity and the presence of other addictive behaviors was moderated by perceived social support, MERS, or DIF, a series of linear regression models was conducted using mean-centered interaction terms. Age and gender were included as covariates in all models.

**Fig. 1:**
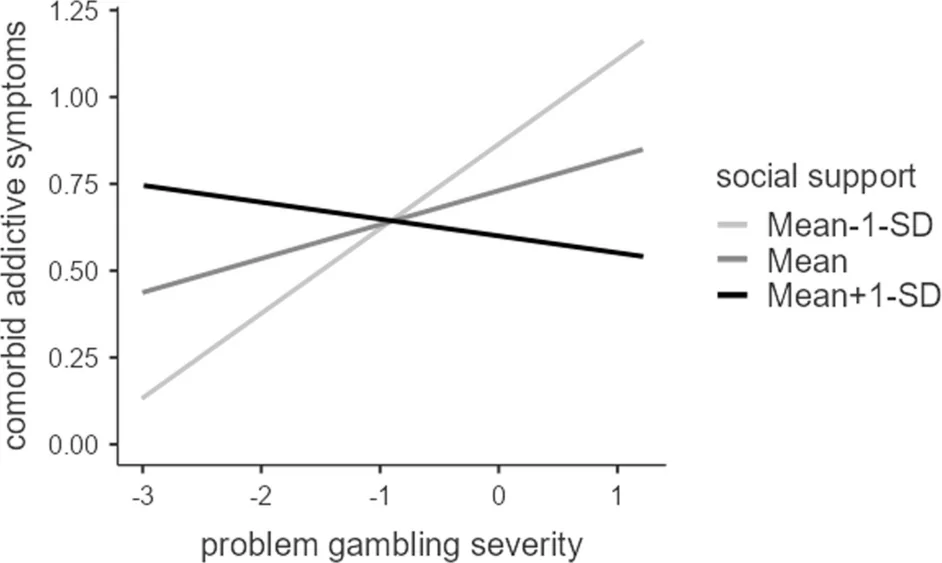
Interaction between problem gambling severity and perceived social support in predicting comorbid addictive symptoms. Lines represent predicted values of comorbid addictive symptoms at low (− 1 SD), mean, and high (+ 1 SD) levels of perceived social support, controlling for age and gender

A significant moderation effect was observed for perceived social support. As shown in Table 3, the interaction between problem gambling severity and social support significantly predicted the presence of other addictive behaviors (β = −1.349, *t* = −2.119, *p* =.038), even after controlling for age and gender. To characterize the nature of this interaction more precisely, we conducted simple slopes analyses at low (− 1 SD), mean, and high (+ 1 SD) levels of perceived social support within a general linear model including problem gambling severity, perceived social support, their interaction term, age, and gender. As shown in Fig. 1, the association between problem gambling severity and comorbid addictive symptoms was significant and positive at low levels of perceived social support (*b* = 0.24, *SE* = 0.09, *p* =.009, β =.45, *η*^*2*^*p* =.11), but non-significant at mean (*b* = 0.10, *SE* = 0.06, *p* =.125) and high levels (*b* = − 0.05, *SE* = 0.10, *p* =.617) of support. Thus, the positive slope relating problem gambling severity to comorbid addictive symptoms was steeper when perceived social support was low and flatter when perceived support was high.

**Table 3:** Moderation analyses testing the interaction between gambling severity and psychological variables in predicting comorbid addictive behaviors (controlled for age and gender)

Outcome	Predictor	*B*	*SE*	*β*	*t*	*p*		95% CI
Comorbid addictive symptoms	(Intercept)	−0.699	1.206		−0.579	.565		[−3.111, 1.713]
	Problem gambling severity	0.768	0.318	1.435	2.413	.019*		[0.132, 1.405]
	Social support	0.017	0.014	0.444	1.197	.236		[–0.011, 0.045]
	Problem gambling severity * Social support	−0.008	0.004	−1.349	−2.119	.038*		[–0.016, –0.001]
	Age	−0.004	0.006	−0.081	−0.667	.507		[–0.016, 0.008]
	Gender	0.006	0.185		0.033	.974		[–0.364, 0.377]
	*R*^*2*^ =.206, *F* (5, 66) = 3.157, *p* =.013*							
Comorbid addictive symptoms	(Intercept)	0.562	1.249		0.450	.654		[–1.932, 3.056]
	Problem gambling severity	–0.252	0.390	–0.452	–0.645	.521		[–1.031, 0.528]
	MERS	–0.007	0.092	–0.030	–0.081	.936		[–0.191, 0.177]
	Problem gambling severity * MERS	0.033	0.029	0.810	1.141	.258		[–0.024, 0.089]
	Age	–0.004	0.006	–0.080	–0.675	.502		[–0.015, 0.007]
	Gender	0.044	0.176		0.248	.805		[–0.309, 0.396]
	*R*^*2*^ =.216, *F* (5, 69) = 3.532, *p* =.007*							
Comorbid addictive symptoms	(Intercept)	0.380	0.551		0.690	.492	[–0.720, 1.480]	
	Problem gambling severity	–0.031	0.171	–0.058	–0.180	.858	[–0.373, 0.311]	
	DIF	0.009	0.023	0.127	0.410	.683	[–0.037, 0.055]	
	Problem gambling severity * DIF	0.007	0.008	0.423	0.960	.340	[–0.008, 0.023]	
	Age	–0.004	0.005	–0.094	–0.831	.409	[–0.015, 0.006]	
	Gender	0.044	0.172		0.255	.799	[–0.299, 0.387]	
	*R*^*2*^ =.242, *F* (5, 70) = 4.159, *p* =.002*							

Gender was coded as 0 = male, 1 = female. *B*, Unstandardized regression coefficient; *β*, Standardized regression coefficient; *SE*, Standard error; *CI*, Confidence interval. *p < 0.05. *MERS*, Maladaptive emotion regulation strategies; *DIF*, Difficulties in Identifying Feelings

In contrast, no significant moderation effects were found for MERS or DIF, as their respective interaction terms with problem gambling severity did not reach statistical significance (*p-*values >.258). Thus, in these models, MERS and DIF did not significantly change the strength of the relationship between problem gambling severity and comorbid addictive symptoms in this sample.

### Mediation Analyses

Mediation analyses were conducted to examine whether MERS or DIF explained the association between problem gambling severity and comorbid addictive symptoms (see Table 4).

**Table 4:** Indirect, direct, and total effects of gambling severity on comorbid addictive symptoms through MERS and DIF (controlled for age and gender)

Mediator	Effect type	*b*	*SE*	*z*	*p*	95% CI
MERS	Direct	0.177	0.055	3.189	.001**	[0.075, 0.303]
	Indirect	−0.048	0.027	−1.764	.078	[−0.124, −0.009]
	Total	0.128	0.060	2.137	.033*	[0.036, 0.247]
DIF	Direct	0.122	0.051	2.386	.017*	[0.010, 0.220]
	Indirect	0.013	0.026	0.523	.601	[−0.038, 0.078]
	Total	0.135	0.060	2.236	.025*	[0.018, 0.242]

*b* = unstandardized regression coefficient*; SE*, Standard error; *CI*, Confidence interval. *p < 0.05, **p < 0.01. *MERS*, Maladaptive emotion regulation strategies; *DIF*, Difficulties in identifying feelings

**Fig. 2:**
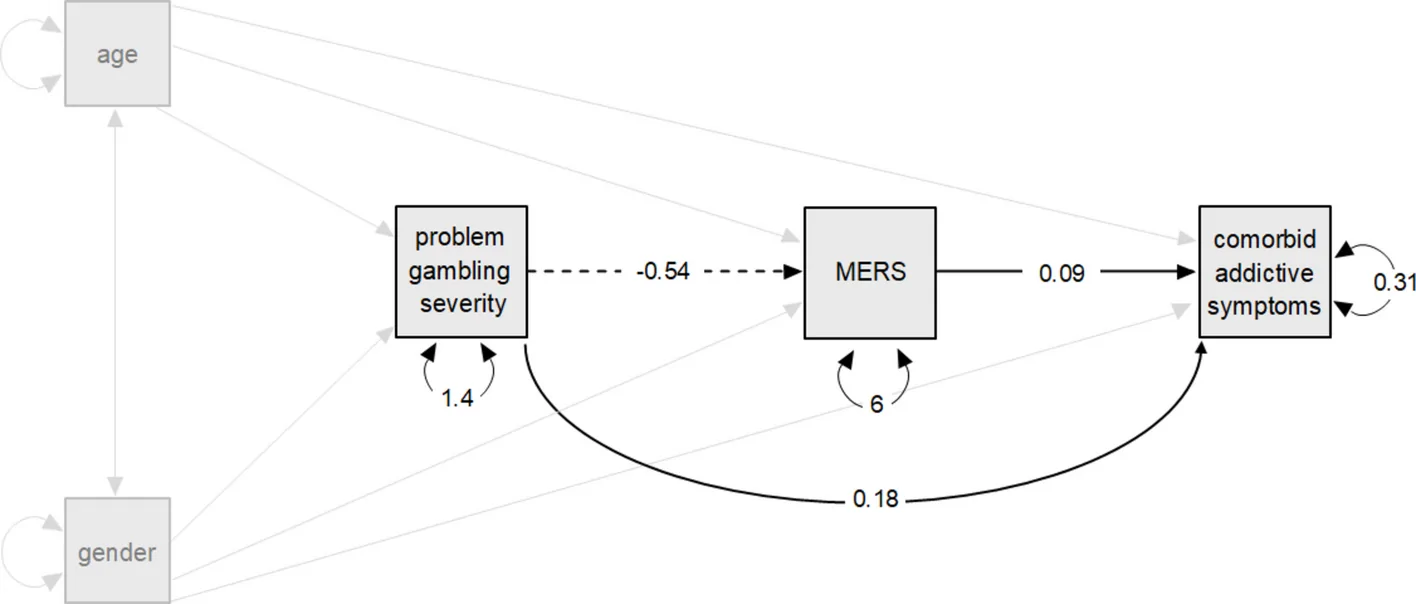
Mediation model testing whether maladaptive emotion regulation strategies (MERS) mediate the association between problem gambling severity and comorbid addictive symptoms, controlling for age and gender. Arrows represent regression paths, with path coefficients shown on each arrow. The curved arrow from problem gambling severity to comorbid addictive symptoms represents the direct effect in the mediation model. The dashed arrow from problem gambling severity to MERS indicates that this path is negative (opposite in sign to the overall positive association between problem gambling severity and comorbid addictive symptoms), consistent with an inconsistent or suppression-type mediation pattern

In the model including MERS as a mediator, the indirect effect of problem gambling severity on comorbid addictive symptoms through MERS showed a *p*-value above the conventional threshold for significance (*b* = −0.048, *z* = −1.764, *p* =.078); however, the 95% confidence interval did not include zero [−0.124, −0.009], suggesting a possible but uncertain mediation effect. Problem gambling severity was negatively associated with MERS, which in turn was positively associated with comorbid addictive symptoms. While these patterns align with partial mediation (as the direct effect remained significant after accounting for MERS: *b* = 0.177, *z* = 3.189, *p* =.001), the tension between the *p*-value and confidence interval calls for cautious interpretation. Notably, the total effect of problem gambling severity was significant (*b* = 0.128, *z* = 2.137, *p* =.033), indicating that MERS may contribute to, but does not fully explain, the association (see Fig. 2).

The model including DIF as a mediator did not yield a significant indirect effect (*b* = 0.013, *z* = 0.523, *p* =.601, 95% CI [−0.038, 0.078]). Although the total (*b* = 0.135, *z* = 2.236, *p* =.025) and direct (*b* = 0.122, *z* = 2.386, *p* =.017) effects of problem gambling severity remained significant, DIF was not significantly associated with problem gambling severity. Therefore, DIF did not meet the conditions to be considered a mediator in this sample.

### Power Sensitivity Analyses

Sensitivity power analyses indicated that, with the present sample sizes, the hierarchical regression model (seven predictors; *N* = 73) had 80% power to detect effects of approximately f^2^ = 0.18 (R^2^ ≈.15). For the moderation models (four predictors; *N* = 66), the minimum detectable effect size at 80% power was approximately f^2^ = 0.22 (R^2^ ≈.18). Thus, the study was adequately powered to detect medium-sized effects in the main regression and moderation analyses.

## Discussion

This study aimed to examine whether problem gambling severity is associated with the presence of other addictive behaviors, and whether AERS, MERS, alexithymia, and perceived social support help explain or moderate this relationship. The findings revealed a modest but consistent association between problem gambling severity and comorbid addictive symptoms. MERS, DIF, and perceived social support each emerged as significant predictors of comorbid addictive symptoms within the regression model. Notably, perceived social support moderates the relationship between problem gambling severity and comorbid addictions, attenuating its strength at higher levels of support. Exploratory mediation analyses suggested a partial mediating role for MERS, although the results should be interpreted with caution given the inconsistency between the *p*-value and the confidence interval. In contrast, DIF did not meet the criteria for mediation, as it was not significantly associated with problem gambling severity. To address concerns about missing data, the main regression, moderation, and mediation models were also re-estimated using full information maximum likelihood (FIML) in R (lavaan), yielding a broadly similar pattern of associations (see Supplementary Appendix S3).

These findings align with previous research emphasizing the transdiagnostic relevance of emotional and interpersonal vulnerabilities across various forms of addiction (e.g., Estévez et al., 2022; Jodis et al., 2023; Marchetti et al., 2019; Marinaci et al., 2021; Palma-Álvarez et al., 2021; Stellern et al., 2023). The positive association between problem gambling severity and comorbid addictive symptoms supports dimensional frameworks of addiction, which posit that shared risk mechanisms (such as emotion dysregulation) may contribute to diverse behavioral manifestations (Cuthbert, 2022; González-Roz et al., 2024; Navas et al., 2017). In particular, the contribution of MERS to the prediction of comorbidity, even after accounting for problem gambling severity, highlights the role of dysfunctional coping in the broader addictive profile (e.g., Burleigh et al., 2019; Cavicchioli et al., 2018).

Interestingly, although MERS significantly predicted comorbid addictive symptoms, their role as a mediator between problem gambling severity and such behaviors was only partially supported. The indirect effect approached significance, and the bootstrapped confidence interval excluded zero, suggesting a possible (albeit tentative) mediation effect. However, this inconsistency between the *p*-value and the confidence interval warrants caution, particularly given the modest sample size, as mediation analyses are often underpowered and may yield inflated Type I error rates in smaller datasets (Koopman et al., 2015). The negative indirect effect was particularly intriguing, as it contrasted with the overall positive association between problem gambling severity and comorbidity. One possible explanation is that individuals with severe gambling problems may increasingly rely on gambling itself as their primary (and often automatic) means of emotion regulation (Neophytou et al., 2021; Ricketts & Macaskill, 2003; Rogier & Velotti, 2018; Weatherly & Miller, 2013), thereby bypassing other maladaptive cognitive strategies. Alternatively, they might underreport MERS due to emotion avoidance or lack of insight, both of which are well-documented cognitive distortions in gambling disorder (Armstrong et al., 2020; Brevers et al., 2013; Riley, 2014). These findings highlight the complex interplay between problem gambling severity and emotion regulation. Moreover, as our data are cross-sectional, alternative temporal or causal configurations cannot be ruled out. It is plausible that difficulties in emotion regulation increase the likelihood of using gambling as a maladaptive coping strategy, and that gambling episodes, in turn, create opportunities or cues for concurrent engagement in other addictive behaviors (e.g., alcohol use in gambling contexts). Conversely, repeated involvement in multiple addictive behaviors may further erode emotion regulation capacities, leading to bidirectional feedback processes. In this sense, our mediation models should be interpreted as exploratory representations of statistical association rather than as evidence for a specific causal pathway.

Regarding the moderating role of social support, the interaction between problem gambling severity and perceived support remained statistically significant even after adjusting for age and gender. The pattern of simple slopes indicated that problem gambling severity was positively associated with comorbid addictive symptoms only at low levels of perceived social support, whereas this association was substantially attenuated and non-significant at mean and high levels of support. This pattern aligns with the buffering hypothesis of social support (Bickl et al., 2024; Cohen & Wills, 1985), suggesting that supportive networks and social environments may protect individuals from the broader spillover of gambling-related distress into other behavioral domains. These findings are consistent with previous research emphasizing the protective role of social support in the context of addictive behaviors (Bickl et al., 2024; Kim et al., 2024; Mancini, 2025; Thomas et al., 2011).

With regard to alexithymia, DIF was positively associated with comorbid addictive symptoms. Notably, in this sample of individuals with diagnosed gambling disorder, DIF did not emerge as a significant moderator or mediator of the relationship between problem gambling severity and comorbidity, suggesting a more limited explanatory role. One possible interpretation is that, within a clinically homogeneous group in terms of gambling problems, individual differences in emotional awareness may be more relevant for understanding variability in additional addictive behaviors.

Several limitations warrant consideration. First, the cross-sectional design precludes causal interpretations. Longitudinal or experimental studies are needed to assess whether emotion regulation and social support precede or result from comorbid behaviors. Second, the fact that the sample consists of clinical patients currently receiving treatment in third-sector organizations may represent a limitation or an important factor to consider when interpreting the results. This context could influence the participants' responses or outcomes, as the therapeutic environment and support services provided by these entities might differ significantly from those found in public or private healthcare settings. Third, all variables were assessed via self-report, potentially inflating shared method variance and reducing accuracy, particularly for constructs like alexithymia. Fourth, the DDF subscale of the TAS-20 (Martínez Sánchez, 1996) showed inadequate internal consistency and was excluded from the main analyses; likewise, the gambling severity dimension of the MultiCAGE CAD-4 (Pérez et al., 2007) showed a reliability coefficient below the conventional threshold of.70, which should be considered when interpreting the results. Future research should consider alternative operationalizations of both emotional expression and problem gambling severity. Fifth, addictive comorbidity was operationalized using a brief screening instrument (MultiCAGE CAD-4), which captures risk indicators and symptoms of multiple addictive behaviors rather than formal comorbid diagnoses. As such, our findings should be interpreted in terms of broader addictive symptomatology, and future studies using structured diagnostic interviews are needed to replicate these results at the diagnostic level. Sixth, our operationalization of comorbid addictive symptoms aggregated seven different domains (alcohol, drugs, Internet, video gaming, compulsive buying, compulsive sexual behavior, and eating-related problems) into a single composite index. This inclusive, transdiagnostic approach was adopted to reduce the number of statistical tests and to capture the overall burden of addictive symptomatology, which was the primary focus of the study and consistent with our limited sample size. However, it also implies that potential behavior-specific associations may have been obscured, and that some of the included domains (e.g., compulsive sexual behavior, eating disorders) are the subject of ongoing debate regarding their classification as addictions. Future research with larger samples and more fine-grained designs should examine whether the mechanisms studied here (emotion regulation, alexithymia, and social support) differentially relate to specific addictive behaviors or to distinct profiles of comorbidity. Seventh, the sample consisted exclusively of individuals undergoing treatment for gambling disorder. While this enhances clinical relevance, it may also limit the generalizability of the findings to individuals not currently engaged in treatment. Treatment-seeking participants may differ systematically from non-treatment-seeking individuals in terms of motivation, psychological insight, or symptom severity, potentially influencing the observed associations. Eighth, our sample size (ranging from 73 to 89 participants) may have limited statistical power, particularly for interaction and mediation models. Sensitivity analyses suggested that the present sample was sufficiently powered (80%) to detect medium-sized effects in the regression and moderation models, but smaller effects may have remained undetected, underscoring the need for replication in larger cohorts (see Statistical Analysis and Results sections for details). Nineth, although re-analyses using FIML under missing data yielded results that were generally consistent with the original models, they also highlight that some effects (particularly the direct association between social support and comorbid addictive symptoms) should be interpreted with appropriate caution (see Supplementary Appendix S3).

Despite these constraints, the present study also has several strengths that we believe add value to the literature. First, it focuses on a well-characterized, treatment-seeking clinical sample of individuals with gambling disorder, which enhances the ecological and clinical validity of the findings compared to studies relying on community or subclinical samples. Second, the study simultaneously examines emotion regulation, alexithymia, and perceived social support within a single analytic framework and tests both their additive and interactive associations with comorbid addictive symptomatology, rather than considering these constructs in isolation. Third, by operationalizing comorbid addictive symptoms using a multi-addiction screening instrument, the study adopts a transdiagnostic perspective that is consistent with emerging dimensional models of addiction. Finally, the combined use of hierarchical regression, moderation, and exploratory mediation analyses provides a nuanced, albeit preliminary, picture of how emotional and interpersonal vulnerabilities may relate to broader addictive comorbidity in individuals with gambling disorder.

In conclusion, this study contributes to the understanding of transdiagnostic processes in behavioral addiction. MERS and social support emerged as significant predictors of broader addictive comorbidity in individuals with gambling problems, while alexithymia showed a more limited role, being only associated with comorbidity in the regression model. These findings highlight the relevance of emotional and interpersonal functioning in both clinical assessment and intervention. In treatment-seeking populations, this suggests that routine assessment should extend beyond gambling severity to systematically include maladaptive emotion regulation strategies, alexithymia, and perceived social support. Patients presenting with high levels of maladaptive strategies and low perceived support may warrant closer monitoring for the emergence or persistence of additional addictive behaviors and could particularly benefit from transdiagnostic interventions that integrate emotion-regulation skills training (e.g., cognitive reappraisal, distress tolerance) with strategies to strengthen and mobilize social networks. Incorporating brief screening tools for multiple addictions (such as the MultiCAGE CAD-4) into intake procedures might help clinicians to detect comorbid addictive symptoms early in treatment and to prioritize integrated, rather than strictly substance- or behavior-specific, treatment plans. Collaboration with family members, significant others, and mutual-help or community-based resources that provide stable interpersonal support may be especially relevant for individuals whose gambling problems occur in the context of broader psychosocial adversity. Preventive and therapeutic efforts aimed at reducing maladaptive coping strategies and strengthening support systems may be valuable in preventing the escalation from isolated gambling behavior to more generalized addictive dysfunction. Given the observed patterns, future studies would benefit from testing moderated mediation models to clarify whether the potential explanatory role of psychological variables (such as MERS) is influenced by contextual factors like perceived social support. This could provide a more nuanced understanding of how emotional and social vulnerabilities jointly contribute to addiction comorbidity. In addition, future research could examine whether these transdiagnostic mechanisms help to refine subtype-based accounts of heterogeneity in gambling disorder, such as those proposed in the Pathways Model (Blaszczynski & Nower, 2002) or Gambling Space Model (Navas et al., 2019).
